# Gene Transfer of Heme Oxygenase-1 Using an Adeno-Associated Virus Serotype 6 Vector Prolongs Cardiac Allograft Survival

**DOI:** 10.1155/2012/740653

**Published:** 2012-10-16

**Authors:** Jacqueline M. Evans, Sonia Navarro, Tomoko Doki, John M. Stewart, Noboru Mitsuhashi, Mary Kearns-Jonker

**Affiliations:** ^1^Department of Anesthesiology Critical Care Medicine, Children's Hospital Los Angeles, Los Angeles, CA 90027, USA; ^2^Department of Cardiothoracic Surgery, Children's Hospital Los Angeles, Los Angeles, CA 90027, USA; ^3^Department of Pathology and Human Anatomy, Loma Linda University School of Medicine, Alumni Hall Room 326, 11021 Campus Street, Loma Linda, CA 92350, USA

## Abstract

*Introduction*. Allograft survival can be prolonged by overexpression of cytoprotective genes such as heme oxygenase-1 (HO-1). Modifications in vector design and delivery have provided new opportunities to safely and effectively administer HO-1 into the heart prior to transplantation to improve long-term graft outcome. *Methods*. HO-1 was delivered to the donor heart using an adeno-associated virus vector (AAV) with a pseudotype 6 capsid and vascular endothelial growth factor (VEGF) to enhance myocardial tropism and microvascular permeability. Survival of mouse cardiac allografts, fully or partially mismatched at the MHC, was determined with and without cyclosporine A. Intragraft cytokine gene expression was examined by PCR. *Results*. The use of AAV6 to deliver HO-1 to the donor heart, combined with immunosuppression, prolonged allograft survival by 55.3% when donor and recipient were completely mismatched at the MHC and by 94.6% if partially mismatched. The combination of gene therapy and immunosuppression was more beneficial than treatment with either AAV6-HO-1 or CsA alone. IL-17a, b, e and f were induced in the heart at rejection. *Conclusions*. Pretreatment of cardiac allografts with AAV6-HO-1 plus cyclosporine A prolonged graft survival. HO-1 gene therapy represents a beneficial adjunct to immunosuppressive therapy in cardiac transplantation.

## 1. Introduction

Chronic graft vasculopathy (CGV), also referred to as “transplant-associated arteriosclerosis,” is a widespread and progressive process characterized by intimal hyperplasia, inflammation, and fibrosis of the graft arterial microvasculature [1]. CGV is a major challenge for heart and other organ transplant recipients [1–4]. Although modern immunosuppressive agents have significantly improved short-term outcomes, long-term outcomes have been less favorable [4]. Ten year survival of adults undergoing heart transplantation is currently at 55% [5]. Moreover, immunosuppressive therapy is hampered by a multitude of potentially life-threatening side effects as well as the need for lifelong compliance and rigorous monitoring [4, 6]. 

Tissue-directed gene therapy in which a therapeutic molecule is produced within the graft itself could reduce the necessity for systemic immunosuppressive agents [7]. Candidate cytoprotective genes include various heat shock proteins (HSPs) [8], B-cell leukemia/lymphoma-2(bcl-2), nitric oxide synthase, interleukins 4 and 10 [9, 10], and transforming growth factor-*β*_1_ (TGF-*β*_1_) [11]. The well-characterized gene heme oxygenase-1 (HO-1, HSP-32) prevents ischemia reperfusion (I/R) injury [12–17], promotes allograft and xenograft survival [1, 18–24], and prevents vascular remodeling [25–27]. HO-1 maintains cellular redox status, preserves vascular patency, and inhibits inflammation and apoptosis [18, 28]. 

Adenoviral vector-mediated administration of HO-1 has been shown to prevent chronic rejection of aortic [29, 30] and cardiac [31] allografts, but clinical use of adenoviral vectors in humans has serious limitations. Wild-type adenovirus is associated with acute respiratory tract infection and administration of adenoviral vectors induces an immune response [32]. In contrast, infection with wild-type adeno-associated virus (AAV) is associated with minimal cellular immune response and is not associated with human pathology [33, 34]. Recombinant AAV (rAAV) vectors have high biosafety profiles and are used in clinical gene therapy trials [34, 35]. rAAV vectors transduce dividing and nondividing cells and persist as extrachromosomal episomal elements, reducing concerns regarding insertional mutagenesis [33]. Infection with rAAV vectors does not increase mutation rates [36] and is associated with long-lasting gene expression [7, 37–39]. 

In the heart, earlier generation rAAV pseudotype 2 vectors have been successfully used to administer HO-1 (rAAV2/HO-1) to prevent I/R injury after myocardial infarction [14, 37–39]. rAAV vectors encoding immunomodulatory or cytoprotective genes may also be beneficial in preventing CGV in patients undergoing heart transplantation [40–44]. The administration of rAAV2 encoding HO-1 modestly prolongs cardiac allograft survival in rats [45]. Later generation vectors, now available, combined with newly identified modifications in vector delivery, may further improve long-term graft outcome. In the study described here, an AAV vector was used to deliver HO-1 to the heart using a later generation pseudotype 6 capsid and coadministration of vascular endothelial growth factor (VEGF) to enhance myocardial tropism and microvascular permeability [46]. Our studies represent the first report comparing graft survival after transplantation of fully and partially mismatched mouse cardiac allografts expressing rAAV6/HO-1. Results from this work may serve as the foundation for future translational studies in which the possibility of HO-1 gene therapy in clinical heart transplantation is explored.

## 2. Materials and Methods

### 2.1. Construction of Adeno-Associated Viral Vectors

HO-1 cDNA was cloned from mouse splenic lymphocytes. Total RNA was used to prepare cDNA. Primers used to clone the HO-1 gene were 5′ AAC TAG CCC AGT CCG GTG ATG GAG C 3′ and 5′ GGG CCA GTA TTG CAT TTA CAT GGC 3′. We used BLAST to confirm that our cloned gene was mouse HO-1 (NCBI accession number, NM_010442). The HO-1 gene was ligated into the rAAV2 backbone, pAAV2:CMV-MCS-SV40pA (kindly provided by Dr. Michael J. Blankinship, University of Washington, Seattle, WA) and the construct was sequenced. The plasmid pAAV2:CMV-HO-1-SV40pA was encapsidated by pA6DG, a packaging/helper plasmid containing a rAAV2 *rep* and a rAAV6 *cap* by the vector core at the University of Washington (Seattle, WA). A rAAV6 vector expressing human placental alkaline phosphatase (rAAV6/AP synthesized by Dr. Jeffry Chamberlain, Department of Neurology, University of Washington, Seattle, WA) was used as a control for our studies.

### 2.2. Western Blot Studies to Confirm Immunoreactivity of Exogenous HO-1

Immunoreactivity of the expressed HO-1 enzyme was confirmed in lysates from transduced minipig kidney (MPK) cells (ATCC, Manassas, VA) and transduced mouse myocardium. MPK cells show no crossreactivity between the antibody for mouse HO-1 and endogenous pig HO-1. To optimize conditions, MPK cells were initially incubated with 2.6 × 10^3^ to 2.6 × 10^5^ vg of rAAV6/ARAP4 and stained for alkaline phosphatase using Sigma SIGMAFAST BCIP/NBTtablets (Sigma-Aldrich, St. Louis, MO). Cells were heated to 65°C prior to staining to inactivate endogenous alkaline phosphatase. MPK cells were transduced with 2.6 × 10^5^ vg of rAAV6/HO-1 for 48 hours and harvested.

For *in vivo *studies, Balb/cJ mice were injected via tail vein with 2 × 10^12^ vg of rAAV6/HO-1, and hearts were harvested at 14d for robust rAAV6-mediated transduction of mouse myocardium [46]. Vector was coadministered with 400 ug/kg of vascular endothelial growth factor (VEGF, RD Systems, Minneapolis, MN) diluted in lactated ringers containing 0.008% mouse serum albumin (Sigma-Aldrich, St. Louis, MO) and 80 IU/kg of sodium heparin to increase microvascular permeability [46].

HO-1 immunoreactivity was measured by homogenizing cells and tissue samples in lysis buffer containing 50 mM Tris-HCl, pH 7.4, 20 mM NaCl, 10 mM KCl, 0.1 mM DTT, 1 mM EDTA pH 8.0 and 0.10% SDS. Protein concentrations were measured by the Bradford method (Pierce BCA Protein Assay Kit, Pierce Technologies, Rockford, IL). Samples were denatured, separated by sodium dodecyl sulfate polyacrylamide gel electrophoresis (SDS-PAGE), and transferred to polyvinylidene fluoride (PVDF) membranes. Immunoreactive HO-1 was detected using a rabbit anti-rat primary antibody (0.5 ug/mL or 1 : 2000 dilution) cross-reactive for mouse HO-1 (Stressgen, Ann Arbor, MI), a polyclonal HRP-conjugated anti-rabbit secondary antibody (0.1 ug/mL or 1 : 10,000 dilution) (Promega, Madison, WI), and a SuperSignal West Pico Chemiluminescent Kit (Pierce Biotechnology, Rockford, IL). A rabbit polyclonal antibody against actin (Santa Cruz Biotechnologies, Santa Cruz, CA) served as a control.

### 2.3. Heme Oxygenase Assay to Confirm Functional Activity of Exogenous HO-1

The functional activity of expressed HO-1 was verified using a paired enzyme assay for HO-1 activity [47]. HO-1 converts heme to equimolar amounts of biliverdin, carbon monoxide and free iron. Bilverdin is converted to bilirubin by an NADPH-dependent biliverdin reductase. In this assay, an unprocessed liver cytosol preparation (105,000 g supernatant from homogenized liver) served as a source of biliverdin reductase. One hundred and forty micrograms of protein from transduced cells or 500 ug of protein from tissue was incubated for 1 hour in the dark at 37°C. Samples were incubated in 50 uM hemin, 0.8 mM NADPH, 2 mM glucose 6-phosphate (G6P), 1.5 mg of liver cytosol, and 0.2 U G6P dehydrogenase (an NADPH generating system). The reaction was terminated with chloroform. Bilirubin generated through the metabolism of hemin by HO-1 was measured spectrophotometrically at 464 nm minus the absorbency at 530 nm (molar extinction coefficient, *ε* = 40 mM^−1 ^cm^−1^). HO-1 activity was reported as picomoles of bilirubin generated per mg protein per hour. A control reaction containing all components minus the test protein (source of HO-1) served as a blank. The spectrophotometric measurements for the blanks were subtracted from all experimental and control samples. 

### 2.4. Distribution Studies

Balb/cJ mice were injected with 2 × 10^11^ vg of rAAV6/ARAP4, a control vector encoding the reporter gene human placental alkaline phosphatase (AP). Hearts were harvested at 11 days. Five micron frozen sections were stained for AP activity using Sigma SIGMAFAST BCIP/NBTtablets (Sigma-Aldrich, St. Louis, MO).

### 2.5. Quantitative PCR to Confirm Persistence of Viral DNA in Cellular Extracts from Genomic DNA

The persistence of viral DNA in cellular DNA extracts from rAAV6/HO-1-transduced hearts was measured at 14 days after treatment of Balb/cJ mice with 2 × 10^12^ or 2 × 10^11^ vg of rAAV6/HO-1/animal. Genomic DNA was extracted (Dneasy Tissue Kit, Qiagen, Valencia, CA) and quantified (Sigma QF Kit, Sigma-Aldrich, St. Louis, MO), against a standard curve. Quantitative PCR (qPCR, Taqman, Applied Biosystems, Pleasanton, CA) was performed using an ABI PRISM 7700 Sequence Detector (Perkin Elmer, Foster City, CA). Primers were designed to target exon junctions to probe for the transgene rather than endogenous DNA. The sequence of the forward and reverse primers and probe were 5′ CAC AGA TGG CGT CAC TTC GT 3′, 5′ GCG GTG TCT GGG ATG AGC TA 3′, and 5′ AGC CTG GTG CAA GAT ACT GCC CC 3′.

### 2.6. Heterotopic Heart Transplantation

Animal experiments were approved by the Children's Hospital Los Angeles Institutional Animal Care and Use Committee. Mice (Jackson Laboratory, Bar Harbor, ME) were male and 6 to 8 weeks old. Transplantation studies involved fully mismatched cardiac allografts (Balb/cJ, H-2d donors and C57Bl/6J, H-2b recipients, Table 1) and partially mismatched cardiac allografts (DBA/2, H-2^d^ donors and B6AF1, H-2^b/a^ or H-2^b, k/d^ recipients). Shown in Table 1, DBA/2 (H-2^d^) mice have a “d” haplotype. Unlike the other strains used in these studies, which are congenic, the B6AF1 strain is an F1 hybrid cross of the B6 (“b” haplotype) and A (“a” haplotype) strains and has a mixed haplotype, H-2^b/a^. The “a” haplotype is a recombinant haplotype and is a cross between the “k” and “d” haplotypes; therefore, H-2^b/a^ can also be written as H-2^b, k/d^. The MHC class Ia locus, K and the MHC class II loci (A*α* and *β* and E*α* and *β*) are “k” while the MHC class Ia loci, D and L and the MHC class III locus, S is “d”. Mice were pretreated with rAAV6/HO-1 or the AP-expressing control vector rAAV6/ARAP4 or vehicle 9 to 14 days prior to heterotopic heart transplantation. Ventricular contractions were assessed daily by palpation. Rejection was determined by cessation of cardiac function and pathology. Allografts were harvested at rejection (absence of palpable ventricular contractions on abdominal exam) or at 100 days after transplantation, divided for biochemical analyses, snap frozen in liquid nitrogen and stored at −80°C. 

### 2.7. Real-Time PCR

Heart tissue was isolated from transplanted animals at the time of rejection or at 100 days after transplantation in animals with long-term surviving grafts. The heart tissue was divided, allowing part of the tissue to be stored in RNA Later (Qiagen, Valencia, CA) and part to be stored snap frozen. RNA was isolated using the RNeasy Fibrous Tissue Kit from Qiagen (Valencia, CA). RNA quality was verified by electrophoresis on 1% agarose gel andcDNA was then prepared using Superscript III (Invitrogen, Carlsbad, CA). Two ug of RNA was used for each cDNA preparation. Real-time PCR was performed using Fast SYBR Green Master Mix (Promega, Madison, WI) and an IQ 5 Multicolor Real-Time PCR Detection System (BioRad, Hercules, CA). The PCR conditions were 94°C for 10 minutes, 94°C for 15 seconds, 60°C for 60 seconds, and 72°C for 30 seconds for a total of 40 cycles. qPCR was run in triplicate. Primers used to identify cytokine expression levels in the heart at the time of rejection or at 100 days in surviving grafts are TNF*α* FWD AGGAGAAAGTCAACCTCCTCTCTG, REV TGGAAGACTCCTCCCAGGTATATG; IL-2 FWD AAGCATTGCCTTCTAGGTCTCC, REV TCAGAGATACACGAGCTGGTT; IFN-*γ* FWD GCCACGGCACAGTCATTGA, REV TGCTGATGGCCTGATTGTCTT; Il-10 FWDCTTACTGACTGGCATGAGGATCA, REV GCAGCTCTAGGAGCATGTGG, Il-4 FWD GGTCTCAACCCCCAGCTAGT, REV GCCGATGATCTCTCTCAAGTGAT; IL-1*β* FWD GAAATGCCACCTTTTGACAGTG, REV TGGATGCTCTCATCAGGACAG; IL-17a FWD TCAGCGTGTCCAAACACTGAG, REV CGCCAAGGGAGTTAAAGACTT; Il-17b FWD GAGTAAAGCCCTACGCTCGAA, REVCTCCTCTTGTTGGACAACCAC; IL-17e/IL-25 FWD ACAGGGACTTGAATCGGGTC, REV TGGTAAAGTGGGACGGAGTTG; and IL-17f FWD TGCTACTGTTGATGTTGGGAC, REV CAGAAATGCCCTGGTTTTGGT.

 GAPDH FWD GGGCATGGACTGTGGTCATGAG, REV TGCACCACCAACTGCTTAGCC. was used as a housekeeping gene.All primers were well documented with validation information provided at the website http://pga.mgh.harvard.edu/primerbank/. Fold changes in gene expression were determined by comparing the Ct for each gene (in triplicate). A minimum of three biological replicates were run in each group before and after transplantation. Data was analyzed using the formula 2^ΔΔCt^ where ΔCt = Ct of our gene of interest-Ct of GAPDH. The results are shown ± SEM.

### 2.8. Statistics

Results were analyzed using ANOVA followed by Bonferroni's Multiple Comparison Test and Kaplan-Meier log-rank analysis (GraphPad PRISM version 5.0, La Jolla, CA). Survival times are listed as mean survival time (MST) ± SEM. Significant differences in gene expression levels were determined by Student's *t*-test, comparing the ΔΔCT of the allograft control group versus the fully or semiallogeneic transplant group treated with AAV6-HO-1 + cyclosporine A. 

## 3. Results

### 3.1. Immunoreactivity, Functional Activity, and Distribution of Expressed HO-1

**Figure 1:**
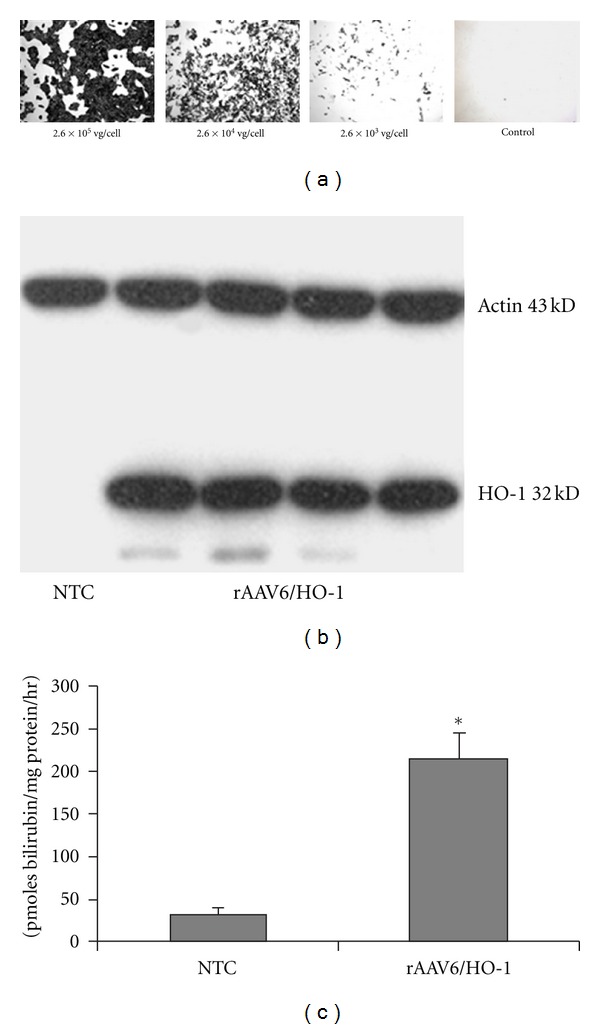
Transduction efficiency *in vitro*. (a) MPK cells transduced at various concentrations by rAAV6/ARAP4, a control expression vector for the reporter gene alkaline phosphatase (AP), were stained with a histochemical stain for AP 72 hours after transduction. (b) Western blot analysis of HO-1 expression by MPK cells transduced with 2.6 × 10^5^ VG/cell of rAAV6/HO-1 for 48 hrs (NTC, nontransduced cells). (c) Enzymatic activity of HO-1 in MPK cells transduced with 2.6 × 10^5^ vg/cell of rAAV6/HO-1. Values are mean ± SEM. *N* = 3 plates NTC cells, *N* = 4 plates rAAV6/HO-1-transduced cells. Each plate was run in triplicate (*P < 0.003 as compared to nontransduced control cells, NTC).

Prior to commencing *in vivo* studies, immunoreactivity and functional activity of expressed HO-1 was demonstrated *in vitro* in rAAV6/HO-1-transduced cultured MPK cells. The concentration of rAAV6/HO-1 for *in vitro* experiments was based on preliminary experiments in which MPK cells were transduced with various concentrations of rAAV6/ARAP4, a control vector encoding the reporter gene human placental alkaline phosphatase (AP) and stained for AP. As shown in Figure 1(a), detectable AP staining was noted after 48-hour incubation with 2.6 × 10^3^ viral genomes (vg) of rAAV6/ARAP4 per cell, while 2.6 × 10^5^ vg/cell showed robust transduction. Incubation of MPK cells with 2.6 × 10^5^ vg/cell using AAV6/HO-1 was associated with production of immunoreactive HO-1 by western blot (Figure 1(b)). There was no detectable HO-1 in nontransduced cells. Western blot findings were supported by measurement of HO-1 functional activity. As shown in Figure 1(c), incubation of MPK cells with 2.6 × 10^5^ vg/cell of rAAV6/HO-1 was associated with a 7-fold increase in functional HO-1 activity versus nontransduced controls.

**Figure 2:**
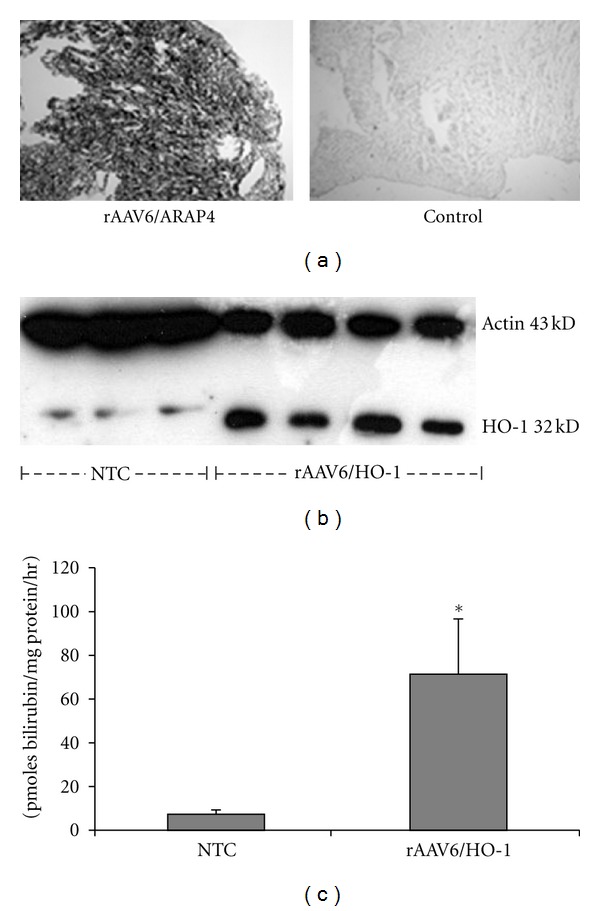
Gene transfer *in vivo*. (a) Alkaline phosphatase staining of Balb/cJ hearts after intravenous injection of 2 × 10^11^ viral genome (VG) particles of rAAV6/ARAP4 as compared to untreated controls. (b) Western blot of hearts from Balb/cJ mice treated with 2 × 10^12^ vg of rAAV6/HO-1 as compared to nontransduced controls (NTC). (c) HO-1 functional activity in hearts from Balb/cJ mice treated with 2 × 10^12^ vg of rAAV6/HO-1 as compared to nontransduced controls (NTC). NTC, *N* = 3, rAAV6/HO-1, *N* = 4. Each sample was run in triplicate (NTC) or quadruplicate (rAAV6/HO-1, *P < 0.05 as compared to nontransduced control cells, NTC).

Expression of HO-1 in myocardium from Balb/cJ mice treated with 2 × 10^12^ vg of rAAV6/HO-1 was markedly increased at 14 days as compared to expression of HO-1 in nontransduced controls (Figures 2(a) and 2(b)). Functional HO-1 activity [47] was 10-fold greater than that observed in hearts from nontransduced controls (Figure 2(c)). Separate experiments were performed to measure the myocardial distribution of vector. Hearts from rAAV6/ARAP4-treated animals showed widespread and homogenous staining for AP activity. There was no detectable staining in hearts from nontransduced control animals. 

### 3.2. Persistence of Viral DNA in Cellular Extracts from Genomic DNA

**Figure 3:**
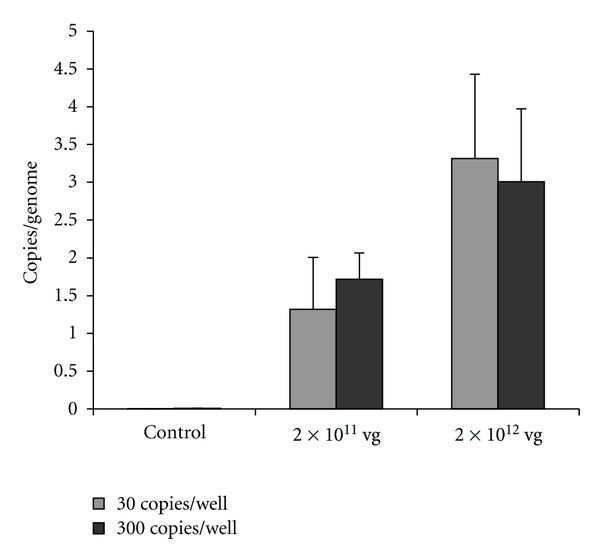
Quantitation of viral genomes in hearts. Heart transduction efficiency ratio was determined based on the amount of vector genome copy numbers in the hearts transduced with 2 × 10^11^ (*n* = 3) or 2 × 10^12^ (*n* = 6) vg of rAAV6/HO-1 as compared to nontransduced control animals. Samples were run at 30 genome copies per well (grey bars) and 300 genome copies per well (black bars). Each animal sample was done in triplicates. Results are mean ± SEM for each sample.

The persistence of viral DNA in extracts of genomic DNA from hearts of Balb/cJ mice transduced with either 2 × 10^11^ or 2 × 10^12^ vg of rAAV6/HO-1 was measured at 14 days. As shown in Figure 3, systemic administration of rAAV6/HO-1 increased levels of viral DNA in the heart identified by Q-PCR in a dose responsive manner. Levels of viral DNA were nondetectable in nontransduced control animals and were elevated in extracts from the hearts of animals receiving 2 × 10^12^ vg as compared to those receiving 2 × 10^11^ vg of rAAV6/HO-1. 

### 3.3. The Effect of rAAV6/HO-1 on Allograft Survival

**Figure 4:**
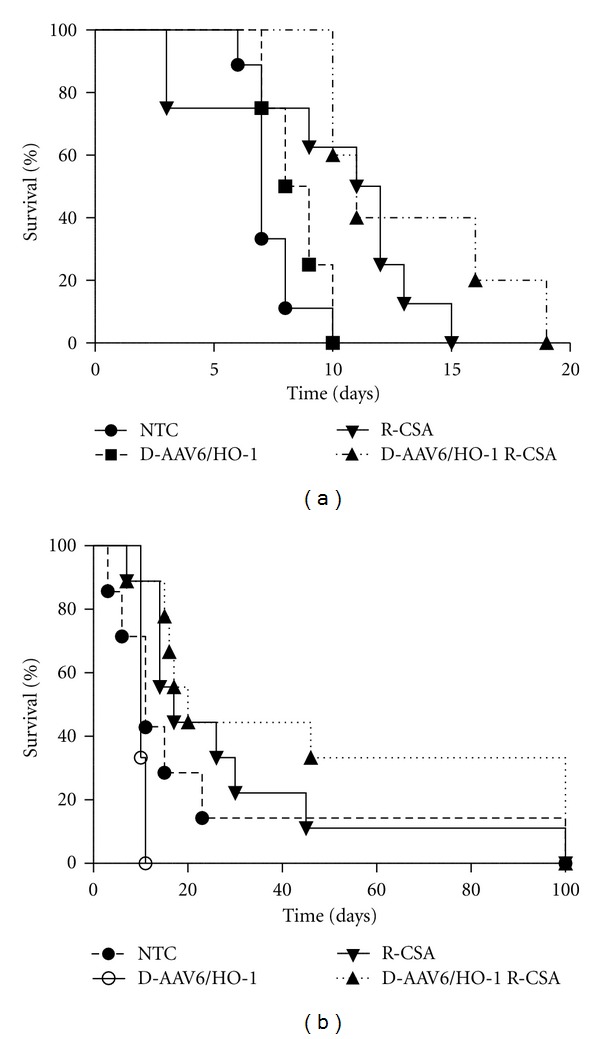
Allograft survival. (a) Graft survival in various treatment groups after transplantation with fully allogeneic hearts. The recipients (R) were male C57BL/6J mice and the donors (D) were male Balb/cJ hearts. Long term graft survival was significantly greater after AAV/HO-1 plus CSA treatment (▲, *n* = 5, *P* = 0.0421) compared with AAV/HO-1 only (■, *n* = 4), CSA treatment only (*▼*, *n* = 8), and controls (●, *n* = 10). (b) In the semiallogeneic model, recipient male B6AF1/J mice were transplanted with donor male DBA/2J hearts. The effect of AAV/HO-1 pre-treatment of the donor and CSA treatment of the recipient was longer-term cardiac allograft survival (▲, *n* = 9, *P* = 0.064) compared with graft survival in the AAV/HO-1-treated group (∘, *n* = 3) or the CSA-treated group without HO-1 (*▼*, *n* = 9). Nontransduced controls are shown as (●, *n* = 7).

The effect of transduction of the donor heart with rAAV6/HO-1 on allograft survival was assessed after transplantation in donor/recipient combinations that were completely or partially mismatched at the MHC loci (Table 1, Figure 4) [48]. Transduced hearts from Balb/cJ mice were transplanted into C57Bl/6J recipients, and graft survival was compared with syngeneic cardiac graft controls. All grafts transplanted into syngeneic controls (C57Bl/6J hearts into C57Bl6/J recipients, *n* = 4) survived for the entire 100-day study period (data not shown) whereas the mean survival time (MST) for grafts from nontransduced control Balb/cJ mice transplanted into C57Bl/6J recipients was 7.4 ± 0.4 days (*n* = 9) (Figure 4). Intravenous (i.v.) administration of 2 × 10^11^ vg of rAAV6/HO-1 eleven days prior to transplantation did not significantly prolong graft survival (MST 8.5 ± 0.6 days, *n* = 4). Treatment of recipients with the immunosuppressant drug CsA to block T-cell activation has been shown to prolong HO-1-mediated cardiac allograft survival in xenograft and allograft models [45, 49]. In subsequent experiments, systemic administration of 2 × 10^12^ vg of rAAV6/HO-1 to donor mice 14 days prior to transplantation was combined with intraperitoneal (i.p.) injection of recipients with 15 mg/kg of CsA on days 0–10. Treatment of recipients with CsA prior to transplantation of rAAV6/HO-1-transduced cardiac allografts significantly prolonged MST (13.2 ± 1.8 days, 55.3% increase, *n* = 5) compared to survival of nontransduced control hearts in untreated recipients (Figure 4(a)). Treatment with CsA prior to transplantation of hearts without genetic modification demonstrated a MST of 9.8 days ± 1.6 days (*n* = 8). 

In order to assess whether AAV/HO-1transduction of the donors combined with CsA-mediated immunosuppression of the recipients would have a greater effect when the MHCs of the donor and recipient were only partially mismatched, hearts from DBA/2 donors were transplanted into B6AF1 recipients (Figure 4(b)). All grafts transplanted into syngeneic control animals (B6AF1 hearts into B6AF1 recipients, *n* = 4) survived for the entire 100-day study period (data not shown) whereas the MST for grafts from nontransduced control DBA/2 mice transplanted into B6AF1 recipients was 24.1 ± 12.9 days (*n* = 7). Transduction of donors with 1 × 10^12^ vg of rAAV6/HO-1 nine days prior to transplantation did not prolong graft survival (MST 10.3 ± 0.3 days, *n* = 3). The combination of i.v. administration of 1 × 10^12^ vg of rAAV6/HO-1 to donor mice 9 days prior to transplantation and treatment of recipients with 15 mg/kg (i.p.) of CsA on days 0–10, however, significantly augmented graft survival to a MST of 46.8 ± 13.8 days, a 94.6% increase in MST compared with controls (*n* = 9). Treatment with CsA alone prior to transplantation of non-transduced allografts did not significantly prolong cardiac graft survival (MST 29.7 + 9.6, *n* = 9).

### 3.4. Intragraft Cytokine Gene Expression

The local cytokine milieu has a role in inducing naïve CD4+ T helper cells to differentiate towards T helper 1 (Th1), Th2, Th17 and regulatory T cells. Skewing the immune response towards Th17 or Th1 and away from T regulatory responses is associated with graft rejection whereas high levels of IL-4 and IL-10 are beneficial to graft survival. Real-time PCR was used to examine cytokine gene expression changes after transplantation of hearts in each of five groups, including (1) hearts that were not genetically modified and represented a donor/host combination that was fully mismatched at the MHC, (2) hearts that were not genetically modified and were partially mismatched at the MHC when compared to their recipients, (3) genetically modified AAV6-HO-1 over-expressing hearts that were fully mismatched at the MHC after transplantation into mice treated with cyclosporine A, (4) genetically modified AAV6-HO-1 hearts that were transplanted in a partial mismatch combination in recipients treated with cyclosporine A and (5) untreated normal hearts. Fold changes in gene expression were determined relative to transplanted hearts in the absence of genetic modification of the donor heart and were also calculated relative to control, untreated hearts. 

Interferon-*γ* and IL-1*β* transcripts were induced at rejection in hearts without genetic modification. Hearts over-expressing HO-1 demonstrated a shift in cytokine expression within the graft. IL-4 and IL-10 transcripts were significantly elevated. IL-10 is a potent anti-inflammatory cytokine whose expression antagonizes Th1 responses via inhibition of antigen presentation and inhibition of interferon gamma production [50]. The cytokines interferon-*γ* and IL-1*β* were significantly downregulated in the AAV6-HO-1 group. 

**Figure 5:**
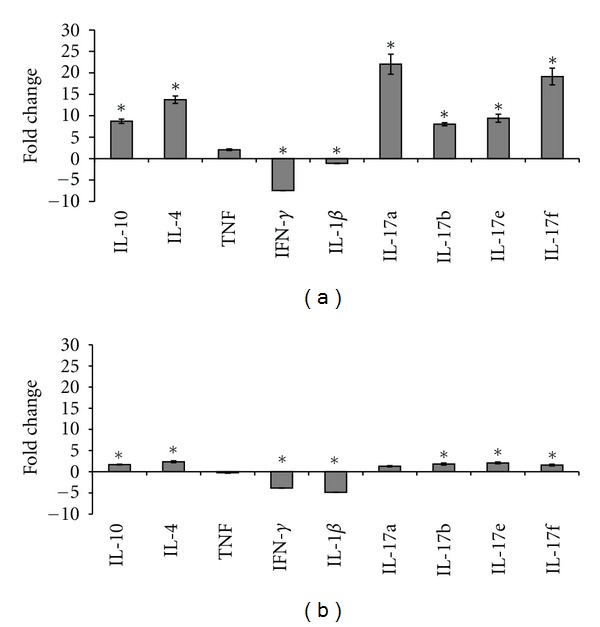
Intragraft cytokine gene expression. Real-time PCR was used to examine cytokine gene expression changes after transplantation of hearts that were (a) fully mismatched at the MHC or (b) partially mismatched at the MHC when compared to their recipients. Interferon-*γ* was not significantly expressed in the transduced hearts but was highly expressed in hearts that were not genetically modified prior to transplantation. IL-17a, IL-17b, IL-17e, and IL-17f were upregulated at rejection (*P < 0.05 comparing ΔΔCT allograft versus HO-1 allograft + cyclosporine (a). IL-4 and IL-10 transcripts were significantly elevated after AAV6-HO-1 transduction of the donor hearts, IL-1*β* was significantly downregulated when compared with nontransduced control allografts. The upregulated genes = ΔΔCT(Experimental)/ΔΔCT(Control) where ΔΔCT = 2^−(GOI−HKG)^. The downregulated genes =  log_2_(ΔΔCT(Experimental)/ΔΔCT(Control)) where ΔΔCT = 2^−(GOI−HKG)^. Samples were run in triplicate and represent a minimum of three biological replicates from each group.

Intragraft cytokine levels in mice rejecting fully allogeneic grafts that were treated with HO-1 could be distinguished from mice accepting their grafts in the semiallogeneic model by an 8–22-fold elevation (*P* < 0.05) in IL-17 transcript levels at the time of rejection (Figure 5). 

## 4. Discussion

HO-1 plays an important role in mitigating I/R injury, preventing transplant arteriosclerosis and prolonging cardiac allograft and xenograft survival [18, 21, 24, 49, 51, 52]. Effective and safe methods of delivery that can be translated into clinical applications will require careful studies to identify vectors with durable expression and a tolerable risk profile [7]. Routes of administration used to deliver genes to donor hearts include intracoronary infusion, intramyocardial and endomyocardial injection, pericardial instillation and systemic *administration* [31, 46, 53, 54]. Recently, Gregorevic and colleagues demonstrated that robust and widespread myocardial gene expression could be achieved after a single systemic injection of the donor with a rAAV6 vector administered with VEGF to increase vascular permeability [46]. We were interested in determining whether HO-1 gene transfer using a rAAV6 construct and this route of administration would improve long-term cardiac allograft survival.

Recombinant adenoviral vectors have been used in numerous gene therapy studies for heart transplantation, but they do not integrate into the host genome and are associated with transient gene expression (~2–4 weeks). Immune responses hamper the efficacy of gene therapy using adenoviral vectors due to elimination of cells expressing viral antigens by cytotoxic T cells and an inability to readminister vector due to the presence of neutralizing antibody arising from previous infections of wild-type adenovirus [7, 32, 55]. 

In contrast, infection with wild-type AAV is safe, effective and induces a minimal cellular immune response [33–35, 56]. These vectors are undergoing continual improvement. Later generation AAV vectors have been reported to be highly efficient at transducing rodent skeletal muscle and myocardium [46, 56, 57]. In the current study, a single systemic dose of a rAAV6 vector encoding HO-1 was used to transduce donor hearts *in vivo. *A rAAV vector with a pseudotype 6 capsid was chosen because of its clinical applicability and strong cardiac tropism as compared to pseudotype 2 vectors [55]. Zincarelli et al. recently compared adeno-associated viral vector serotypes 1–9 and reported that AAV6 was most efficient for high level transduction of the mouse myocardium in a tissue-specific manner [56]. Our report is the first use of a rAAV6 vector overexpressing HO-1 in a cardiac model in which survival of fully or partially mismatched allografts is compared. Braudeau and coworkers observed enhanced survival of cardiac allografts after intragraft (13.3%), intramuscular (62.5%), and i.v. (80%) administration of an adenoviral vector encoding HO-1 in rats completely mismatched at the MHC loci [31]. Tsui and coworkers used an AAV2 vector to transduce cardiac allografts in rats partially mismatched at the MHC [45] and achieved 60% survival of transduced grafts. Survival was increased to 89% by additional treatment with CsA [45]. In our study, rAAV6-HO-1 pretreatment of the donor heart, combined with immunosuppression of the recipient, prolonged allograft survival by 55.3% when donor and recipient were completely mismatched at the MHC and by 94.6% when donor and recipient were partially mismatched at the MHC. 

Prolonged graft survival was associated with intragraft changes in cytokine expression. We and others have shown that elevated levels of IL4 and IL10 can be identified in long-term surviving grafts, but the contribution of IL-17 subsets to graft survival/rejection after cytoprotective gene transfer has not, to our knowledge, been reported previously. IL-17 has recently been identified as a major contributor to graft rejection as demonstrated in several models, including knockout mice (reviewed in [58–60]). There are six IL-17 family members (IL-17 A-F). In our model, IL-17a, b, e and f were notably elevated at the time of graft rejection. IL-17a and IL-17f have been reported to induce pro-inflammatory cytokines and neutrophil infiltration [61]. The proinflammatory response was similarly elevated at rejection in our study. Transcripts encoding all subsets of IL-17 remained unchanged relative to the native heart in long-term surviving grafts overexpressing HO-1. 

Our data support the conclusion that pre-treatment of cardiac allografts with AAV6-HO-1 provides an added benefit and significantly prolongs graft survival in cyclosporine A-treated recipients. The inability to significantly prolong graft survival via HO-1 overexpression alone in the current study may be due to the greater stringency of the mouse as compared to the rat MHC system. 

## Figures and Tables

**Table 1a:** (a) Complete mismatch

		Class Ia	Class II				Class III	Class Ia	
Strain	Haplotype	K	Ab	Aa	Eb	Ea	S	D	L
Balb/cJJ	d	d	d	d	d	d	d	d	d
C57BL/6J	b	b	b	b	b	Null	b	b	Null

**Table 1b:** (b) Partial mismatch

		Class Ia	Class II				Class III	Class Ia	
Strain	Haplotype	K	Ab	Aa	Eb	Ea	S	D	L
DBA/2J	d	d	d	d	d	d	d	d	d
B6AF1/J	b/a	b/k	b/k	b/k	b/k	Null/k	b/d	b/d	b/d

## Abbreviations

rAAV6 vector: Recombinant adenovirus-associated serotype 6 vector
HO-1: Heme oxygenase-1.
